# Assessing the efficacy of the healthy eating and lifestyle programme (HELP) compared with enhanced standard care of the obese adolescent in the community: study protocol for a randomized controlled trial

**DOI:** 10.1186/1745-6215-12-242

**Published:** 2011-11-16

**Authors:** Deborah Christie, Lee Hudson, Anne Mathiot, Tim J Cole, Saffron Karlsen, Anthony Kessel, Sanjay Kinra, Steve Morris, Irwin Nazareth, Ulla Sovio, Ian CK Wong, Russell M Viner

**Affiliations:** 1 General and adolescent paediatrics unit, UCL Institute of Child Health, 30 Guilford Street, London, WC1N 1EH, UK; 2 MRC Centre of Epidemiology for Child Health, UCL Institute of Child Health, 30 Guilford Street, London, WC1N 1EH, UK; 3 Department of Epidemiology and Public Health, University College London, 1-19 Torrington Place, London, WC1E 6BT, UK; 4 Honorary Professor, Department of Social & Environmental Health Research, Faculty of Public Health & Policy, London School of Hygiene & Tropical Medicine, 15-17 Tavistock Place, London WC1H 9SH, UK; 5 Department of Non-Communicable Disease Epidemiology, London School of Hygiene and Tropical Medicine, Keppel Street, London, WC1E 7HT, UK; 6 Department of Primary Care & Population Health, University College London Medical School (Royal Free Campus), Rowland Hill Street, London, NW3 2P, UK; 7 Department of Medical Statistics, London School of Hygiene and Tropical Medicine, Keppel Street, London, WC1E 7HT, UK; 8 Department of Pharmacology and Pharmacy, Li Ka Shing Faculty of Medicine, University of Hong Kong

## Abstract

**Background:**

The childhood obesity epidemic is one of the foremost UK health priorities. Childhood obesity tracks into adult life and places individuals at considerable risk for diabetes, cardiovascular disease, liver disease and other morbidities. There is widespread need for paediatric lifestyle programmes as change may be easier to accomplish in childhood than later in life.

**Study Design/Method:**

The study will evaluate the management of adolescent obesity by conducting a Medical Research Council complex intervention phase III efficacy randomised clinical trial of the Healthy Eating Lifestyle Programme within primary care. The study tests a community delivered multi-component intervention designed for adolescents developed from best practice as identified by National Institute for Health and Clinical Excellence. The hospital based pilot reduced body mass index and improved health-related quality of life.

Subjects will be individually randomised to receiving either the Healthy Eating Lifestyle Programme (12 fortnightly family sessions) or enhanced standard care. Baseline and follow up assessments will be undertaken blind to allocation status. A health economic evaluation is also being conducted.

200 obese young people (13-17 years, body mass index > 98^th ^centile for age and sex) will be recruited from primary care within the greater London area.

The primary hypothesis is that a motivational and solution-focused family-based weight management programme delivered over 6 months is more efficacious in reducing body mass index in obese adolescents identified in the community than enhanced standard care.

The primary outcome will be body mass index at the end of the intervention, adjusted for baseline body mass index, age and sex.

The secondary hypothesis is that the Healthy Eating Lifestyle Programme is more efficacious in improving quality of life and psychological function and reducing waist circumference and cardiovascular risk factors in obese adolescents than enhanced standard care assessed at 6 and 12 months post baseline assessment.

Improvement in quality of life predicts on-going lifestyle change and maximises the chances of long-term weight reduction. We will explore whether improvement in QOL may be intermediate on the pathway between the intervention and body mass index change.

**Trial registration:**

ISRCTN: ISRCTN99840111

## Background

The obesity epidemic in children and young people is one of the foremost clinical and public health priorities in the UK [1]. The UK prevalence of childhood obesity has > tripled since the 1980s. The UK Government's Foresight Report estimates 7-10% of children 5-15 years are obese using the conservative definitions of the International Obesity Task Force [2]. This is predicted to rise to 14% by 2025 [1]. 1-3% of adolescents 11-15 years have a BMI ≥ 3.5 standard deviations above the mean, [2] equivalent to adult morbid obesity (BMI ≥ 40 kg/m^2^).

Obesity in childhood is associated with current and future morbidity [3]. Childhood obesity and cardiovascular risk factors track strongly through adolescence into adult life [4,5]. Around 70% of obese children become obese adults [6] and childhood obesity increases later cardiovascular and all-cause mortality [7,8]. Other comorbidities of adult obesity include metabolic syndrome, type 2 diabetes, a range of cancers and psychosocial dysfunction.

Current obesity comorbidity in children is high. Overweight and obese children report dramatically lower quality of life (QOL) than healthy-weight children [9-11]. Over 70% of obese adolescents have ≥ 1 co-morbidity such as insulin resistance, dyslipidaemia or hypertension; 30% have multiple co-morbidities [12]. Cardiovascular dysfunction related to obesity is identifiable in childhood [13,14].

The UK's cross-government strategy on obesity, Healthy Weight, Healthy Lives: A Cross-Government Strategy for England (January 2008), places a strong focus on children and outlines multidimensional strategies for primary and secondary prevention within and outside the NHS. Each Primary Care Trust (PCT) has an obesity strategy. As part of this, the National Child Measurement Programme (NCMP) was established in 2005 to weigh and measure children in Reception (aged 4-5 years) and Year 6 (10-11 years). The findings are used to inform local planning of services for children and gather population-level surveillance data.

Childhood obesity is an identified national priority for high quality care for the National Health Service in England. The recent Child Health Strategy Healthy Lives, Brighter Futures (2009) identified the reduction of childhood obesity as a key part of improving the health and well-being of children and young people [15].

The NHS has responsibility for treatment of the 7-10% of children already obese. The UK National Institute of Health and Clinical Excellence (NICE) guidance in 2006 outlined a Childhood Obesity Pathway (COP) covering best practice management in primary and secondary care [16].

The development of an effective childhood obesity pathway is an important part of improving child health and reducing NHS burden in the short and long term. However, while there has been considerable public health and research attention focused appropriately on primary and secondary prevention, [1] the management of those 7-10% of children already obese has been relatively neglected. There is a lack of data on the application of the small evidence base for obesity management [17] which constrains the development of services for obese children.

NICE outlined optimal therapy as multicomponent lifestyle modification programmes, however there is a lack of evidence for effective interventions to tackle obesity. Systematic reviews have found few quality trials, principally involving small numbers of primary school aged children, carried out in academic tertiary care centres with highly specialised staff involving white, middle class, motivated families [18]. The applicability and generalisability of these studies is therefore limited, and the reviews conclude that there is an need for quality trials of adequate power to be carried out in samples that are representative of the population at large, where process evaluation has been addressed and appropriate lifestyle tools applied.

Whilst lifestyle modification programmes are the most effective available treatment for childhood obesity, [16] few include those over 12 years of age and none specifically target older adolecsents > 15 years. Dramatic cognitive and psychosocial changes during adolescence make childhood programmes inappropriate for adolescents. Currently adolescents are therefore either excluded from weight management programmes or must join inappropriate child or adult programmes.

The HELP programme has been developed to answer this gap in the childhood obesity pathway. It is a novel intervention for adolescents 13-17 years based upon best evidence. The HELP programme was developed from best practice as identified by NICE [16]. HELP works as a multi-component intervention that focuses on enhancing motivation to change and developing self-efficacy and self-esteem rather than focusing on weight. Pilot data on BMI change in the HELP programme from 20 subjects aged 13-17 years showed a mean BMI reduction 1.7 kg/m^2^, equivalent to 0.4SD effect size in the UK population.

## Method

### Design

HELP is a MRC complex intervention phase III efficacy randomised clinical trial. Subjects will be individually randomised to receiving either the HELP intervention or enhanced standard care for 6 months. Baseline and follow up assessments will be undertaken blind to allocation status. An economic evaluation is also being conducted.

### Ethical consideration

Study information packs will be sent to all potentially eligible young people after the initial screening telephone call. Families will thus have an opportunity to read the information sheets more than 24 hours before attending for their baseline study visit. At the baseline visit, written informed consent will be obtained by one of the research team from all young people and from one responsible parent.

There are likely to be few risks relating to participating in a trial of a pragmatic community-based lifestyle modification intervention related to obesity. The intervention is based around motivational and solution-focused psychological approaches. Motivational Interviewing (MI) is a psychological approach developed for conditions where subjects are reluctant to enter treatment and aims to increase motivation to change behaviours. The approach is based on the degree to which behaviour change is important to an individual, their confidence in their ability to achieve behaviour change and the degree to which change is a priority. Solution-focused therapy (SFT) views the patient rather than the professionals as the expert in order to identify "what works" e.g. identifying "what helped" and has been suggested as a useful technique in weight management.

The professional working with the young person and family helps young people explore changing their lifestyle to be healthier, and works with existing strengths and motivation to do this. The control arm consists only of a single educational session around healthy eating and healthy activity.

### Possible Risks

The potential risks in the study are minimal. There is the potential for young people to become distressed during the assessment as a result of the brief physical examination, blood pressure measurement and completion of psychological and lifestyle questionnaires. We anticipate potential psychological issues will be minimal, as the intervention is NOT designed to explore deep psychological issues or achieve deep psychological insights, but rather to build on the strengths, resources and known abilities of young people and their families. In our pilot work with approximately 20 young people and families, no young people complained of increased distress from taking part in the intervention. The providers will be trained to minimise any possible distress and deal with any distress that may occur and will have access to clinicians with extensive clinical experience.

### Burdens

a) Assessment: The study requires a brief physical examination and venesection at baseline and at week 26. EMLA cream will be offered to reduce pain.

b) Participation in the intervention (i.e. attending for 12 × 45 minute sessions over 6 months) or control (1 × 40 minute session over 6 months).

The West London Research Ethics Committee 3, part of the National Research Ethics System (NRES) approved the study.

### The Study intervention

The HELP programme is a solution-focused and motivational weight management programme. Young people and families will attend twelve 40-45-minute sessions over 6 months. A key aspect of the programme is the use of motivational interviewing and solution-focused approaches to increase engagement and concordance with the 4 programme components.

The 4 components are:

a) modifying eating behaviours and encouraging regular eating patterns

b) decreasing sedentary behaviour and increasing lifestyle and programme activity

c) reducing intake of energy dense foods, and increasing healthy nutritional choices

d) addressing emotional eating triggers.

Providers receive training in specific motivational interviewing techniques and the use of solution focused questioning. A manual is used to enable delivery in a standardised manner by all the providers. Self assessment checklists, live observations and audio recordings of a quasi-random sub-sample of sessions will be used to record adherence to the programme using a Fidelity Adherence Scale to assess fidelity to delivery of session components and adherence to the psychological model. Structured and semi-structured interviews will be administered to providers, young people and their parents to assess how acceptable the programme was, ease of delivery, participation and influence on weight, quality of life, self management, emotional, behavioural and family functioning. In addition administrative records will be used to review attendance and service use.

### The Control arm

Controls will be offered enhanced standard care, defined as one prewritten standardised educational session delivered to young people within three months of recruitment. The Session will be delivered by a practice nurse at the young person's general practice. The Session will be expected to last 40 minutes and will incorporate standard Department of Health guidance and published information on obesity. The session will provide information addressing eating behaviours, healthy activity levels and healthy eating patterns. No training in delivery style will be offered. Where a practice nurse is not available, a trained nurse practitioner will be provided by the study team. Fidelity monitoring will be used on a random subsample of sessions to ensure fidelity of information giving and monitor whether delivering practitioners use motivational or other techniques which are not part of the control session.

### Identification of eligible patients

We will recruit subjects from primary care within the Greater London area. Data from the Foresight Report suggest that approximately 7-8% of 13-16 year olds are obese using a highly conservative definition of obesity (BMI > IOTF threshold) [1,2]. Greater London contains 415,000 young people 13-17 year old (ONS 2008); conservatively 7% are obese, therefore 29,000 are potentially eligible. We will recruit 200 i.e. 0.6% of this population. Note that we will use a slightly less conservative definition of obesity consistent with the NICE guidelines [16] (BMI > 98^th ^BMI centile on the UK 1990 growth reference [19]) to define our eligible subjects, so that the potential pool of subjects is wider than this.

We will identify the young people from the following sources within the PCTs:

1. Primary care networks. We will advertise and recruit subjects through:

i. School nurses in all secondary schools in the region. Nurses will be ask to identify and refer eligible subjects known to them or presenting to them as being obese who may benefit from the intervention.

ii. GP practices. These will be contacted through the PCT networks and through the local Primary Care Research Network (PCRN). We will:

a. ask GPs to identify and refer eligible subjects known to them or presenting to them during the study period as being obese who may benefit from the intervention.

b. ask practice administrators to identify potentially eligible young people through practice databases, and send them study information packs asking them to contact the research team if they are interested in participating.

iii. Other community professionals. Other community based professionals such as dieticians will be asked to identify eligible subjects known to them or presenting to them during the study period as being obese who may benefit from the intervention.

2. Advertising. We will place adverts for the study in community and social media, newsletters and halls. Adverts will describe the study and will provide contact details for the research team.

We aim to recruit 200 subjects between Month 4 and Month 30, i.e. 26 months, being approximately 88 working weeks i.e. 10 subjects per month.

Initial recruitment will be attractive for young people as everyone will receive a comprehensive health assessment and evidence-based advice on weight management. Those young people randomised to the intervention arm will be able to attend sessions delivered locally and at a convenient time after school. Recruitment compensation (a £20 i-tunes or high street store voucher) and travel costs to attend sessions will be provided for all participating young people and families.

### Determining eligibility for the study/Exclusion criteria

Eligibility (see table 1) for the study is determined in two stages. Young people and families who respond to mailed PCT invitations, to advertisement or are referred by school nurses or primary care professionals will be contacted by the research team and complete an initial screening telephone call. Where telephone contact cannot be made, families and young people will be invited by letter or email to attend a face to face screening interview with one of the research team. Young people identified as eligible will be sent information packs including information sheets for young people and families and invited to attend a baseline assessment at the NIHR Great Ormond St Hospital Clinical Research Facility (CRF) by the medical research fellow and the CRF nurses. This appointment is used to formally assess eligibility through anthropometry, psychological questionnaires and venepuncture. Formal written consent will be obtained at this point in the study. If the parent does not speak adequate English, an interpreter will be provided to obtain consent if the young person was under 16 years.

**Table 1:** Study inclusion and exclusion criteria

Inclusion criteria	
1	Young people aged 13-17 years living in Greater London

2	Obese, defined as BMI > 98^th ^centile for BMI using the UK 1990 growth reference [19].

Exclusion criteria	

1	Participants with significant mental health problems or undergoing mental health treatment.

2	Other chronic illness, known secondary obesity, monogenic obesity syndrome or use of medications known to promote obesity. Young people with asthma will be included in the study so long as they have not had more than one course of oral steroids in the preceding 3 months (where course is less than or equal to 5 days), or are on more than the first starting dose of inhaled steroids as per British Thoracic Society Guidelines.

3	Participants with significant learning disability

4	Participants with lack of command of English sufficient to render them unable to participate effectively in the planned intervention. The great majority of eligible young people from black or minority ethnic groups in this population have good command of English. Given the importance of standardising the intervention, it will not be possible to use interpreters to enable parents with poor English to participate. To allow as many young people as possible to participate while maintaining the external validity of the study, we will allow another relative with good English to participate alongside the young person (if they wish it).

5	Participation in behavioural weight management programmes in the past 12 months. This does not include participation in commercial programmes such as Weight Watchers.

6	Young people with BMI > = 40 kg/m2. We exclude this group as they are unlikely to benefit from a community based intervention such as HELP.

Eligible subjects will then be randomized and a member of the study team will be allocated to contact them to arrange times for them to attend either the intervention of control sessions.

### Randomisation

Randomisation will be undertaken independently of the investigators by the Health Services Research Unit (HSRU) University of Aberdeen. Randomisation to the HELP Trial is performed using a secure website. A minimisation protocol will be used for randomisation [20]. Allocation to treatment will ensure balance in respect of one key prognostic variable (sex). The program design has been successfully used by HSRU in several trials and incorporates the use of a library of stored procedures to calculate the appropriate treatment based on subgroup totals stored in SQL Server 2005 data tables. Allocation will be 1:1 to Intervention:Control.

The program will generate a 5-digit Study ID. The first 2 digits identify the recruiting centre (Centre IDs will be allocated when they join the study) and the last 3 identify the individual patient recruited. This Study ID number can then be used subsequently to maintain patient anonymity. At the time of randomisation the HSRU user will select values for the minimising variable Gender, then click on the 'Randomise' button. The study number and the allocated treatment will be displayed and this data then conveyed to the Trial Manager.

### Assessment

Assessments will be undertaken at the NIHR Great Ormond St Hospital Clinical Research Facility (CRF) by the medical research fellow and the CRF nurses using a standardised protocol who are not involved in delivery of the intervention and who will be blind to the randomisation status of the participants.

The patient's GP will be informed if the pathology results are abnormal or concerning.

Outcomes will be measured at Baseline (Week 0), Week 13, end of intervention (Week 26) and 6 months post intervention (Week 52) (see table 2).

**Table 2:** Timing and content of study assessments

	Week 0	Week 13	Week 26	Week 52
Anthopometry (BMI, waist)	X	X	X	X

Motivation	X		X	X

Quality of life measure	X		X	X

Blood pressure	X	X	X	X

Venepuncture	X		X	

Psychological function	X		X	X

Accelerometry	X		X	X

Health economic data EQ5D	X	X	X	X

### Outcomes

A. Primary outcome

The primary outcome is the BMI (kg/m^2^) at the end of the intervention (Wk 26), adjusted for baseline BMI, age and sex.

While it is common to use BMI standard deviation score (SDS) to express change in BMI in children and adolescents, one of us (Cole) has recently shown that change in "raw" BMI is the best outcome measure in obese adolescents, as the skewness in the BMI distribution means that the same change in kg/m^2 ^will produce a smaller change in BMI SDS the more obese the subject is [21].

B. Secondary outcomes

1. Health-related quality of life (HR-QOL) will be assessed using two measures.

i. The Pediatric Quality of Life Inventory (PedsQL) is one of the most widely used measures of HR-QOL in children and adolescents and has proven validity in clinical and population samples [22,23]. It has four core scales (physical, emotional, social, and school), two broad domain scores (physical and psychosocial functioning) and a total score. The measure has previously been used extensively in studies of obese children in community and clinical samples [24,25].

ii. The Impact of Weight on Quality of Life (IWQOL)-Kids [26] is a 27-item instrument consisting of four scales: physical comfort (six items), body esteem (nine items), social life (six items), and family relations (six items). It has excellent psychometric properties [26].

2. Anthropometric measures (completing using a standardised protocol):

i. BMI (6 months after end of intervention)

ii. Waist circumference

iii. Non-invasive measurement of fat mass/fat percentage by Tanita 418 bioimpedance scales

3. Psychological factors:

i. Eating Attitudes Test, a validated self-report 26 item checklist of issues relating to eating disorder and body image [27].

ii. Rosenberg Self-Esteem Scale: the most widely used measure of global self-esteem and has been determined to be valid and reliable for adolescents [28].

iii. Psychological health will be measured using the online version of the Development and Well-being Assessment (DAWBA) interview [29]. Young people and families will be asked to complete the DAWBA confidentially on-line either before or at the assessment. This is a semistructured package of questionnaires and rating techniques designed to generate ICD-10 and DSM-IV psychiatric diagnoses for 5-17 year olds. It identifies common emotional, behavioural and hyperactivity disorders, and has been validated in the recent UK national mental health study of children and adolescents [30].

4. Lifestyle

i. Questions on smoking activities, meal skipping, sociable eating and frequency of 5 fruit a day intake.

ii. We will use an Actigraph accelerometer [31] 7-day measurement once in each subject to calibrate their activity diary.

5. Cardiometabolic risk factors.

i. Fasting insulin and glucose. Insulin resistance will be estimated using the Homeostatic Model Assessment (HOMA) [32].

ii. Fasting lipids: total cholesterol, HDL-cholesterol, LDL-cholesterol, triglycerides and total cholesterol/HDL-C ratio.

iii. Peripheral blood pressure measured accurately using electronic Dinamap.

6. Health economic data:

A prospective economic evaluation will be conducted alongside the trial with the aim of estimating the cost-effectiveness of the HELP intervention versus enhanced standard care.

Preference-based HR-QOL will be characterized using the EQ-5D and will be administered to participants at baseline, month 3, month 6, and month 12. Information on resource utilisation and on the costs associated with the HELP and enhanced standard care interventions will be obtained using observational research methods and through interviews. The resource utilisation of the adolescents during the trial will be informed using surveys sent to participants at months 0, 6 and 12. Current UK unit costs will be applied to each resource item to value total resource use in each arm of the trial.

### Power and sample size

We have powered our study to detect an 0.5 SD effect size for the primary outcome. This will also detect the same effect size for secondary outcomes. Our pilot data identified a likely effect size of 1.7 kg/m^2 ^reduction in BMI. This is equivalent to 0.6 SD given evidence from our pilot study and from other interventions [18] that the SD of change in adolescent BMI is approximately 2.0 kg/m^2^. Lifestyle modification programmes in obese children result in improvements in HR-QOL of 0.3-0.5 SD [33,34].

Sample size: 126 subjects is sufficient to detect an effect size of 0.5 at 80% power and 5% significance. This sample will be inflated to account for the clustering due to any persistent therapist effects [35]. Using a therapist cluster size (C) of 10 subjects and an assuming an ICC of 0.025, we will require an inflation of our existing sample size to (1226 × (1 + (C-1)*0.025)) = 155. We will recruit 200 subjects to allow for 20% drop-out.

### Statistical analysis

Main analysis of primary outcome: Analyses will be undertaken by the programme statistician, supervised by Cole. An intention to treat analysis will be undertaken for the primary outcome. A linear regression model that accounts for clustering effects will be fitted. The main analysis will incorporate correction for missing data. The model can either be fitted using an EM algorithm or MCMC approach. This analysis will be performed adjusted for baseline BMI, age and sex. The biases caused by missing 3 and 6 months outcome data will be addressed through multiple imputation by methods that utilise the number of attempts to achieve response [36] as well as other sensitivity analyses for informative missingness [37]. We will undertake a complier average causal effect (CACE) analysis to identify the difference in mean outcome amongst compliers. CACE recognizes the initial randomization and thus overcomes the problems faced by per-protocol and on-treatment analyses.

Secondary analyses of primary outcome:

a. The main analysis will be repeated without correction for missing data, e.g. using complete cases analysis (intention to treat), adjusted for baseline BMI, age and sex.

b. Complier average causal effect (CACE) analysis to identify the difference in mean outcome amongst compliers, accounting for missing data, adjusted for baseline BMI, age and sex.

c. CACE analysis without correction for missing data, adjusted for baseline BMI, age and sex.

d. Sensitivity analyses for informative missingness.

In this study, compliance for the intervention arm is defined as fulfilling the following criteria: 1) attendance to session one, 2) attendance to at least one session in each of the four modules of the intervention, and 3) attendance to a minimum of eight of the twelve sessions in total. Participants in the intervention arm failing to fulfil one or more of these criteria are classified as non-compliers.

Main analysis of secondary outcomes:

In addition to secondary outcomes listed above, BMI at 6 months after end of intervention (Week 52) is included as a secondary outcome. Similarly to the primary outcome analyses, an intention to treat analysis will be applied. A linear or logistic regression model that accounts for clustering effects will be fitted for continuous and binary outcomes, respectively. For ordinal outcomes, such as LIKERT scale outcomes, an ordinal logistic regression model will be fitted. The main analysis will incorporate correction for missing data.

Secondary analyses of secondary outcomes:

a. The main analysis will be repeated without correction for missing data, e.g. using complete cases analysis (intention to treat), adjusted for baseline BMI, age and sex.

b. CACE analysis to identify the difference in mean outcome amongst compliers, accounting for missing data, adjusted for baseline BMI, age and sex.

c. CACE analysis without correction for missing data, adjusted for baseline BMI, age and sex.

Software: Depending on the choice of implementation, standard software for generalised linear models, e.g. STATA (to implement EM algorithm) or WinBUGS (to implement MCMC) will be used for the main analyses.

Reporting of the statistical analyses will follow CONSORT guidelines.

### Economic evaluation

The economic evaluation will be conducted from the public services (NHS, education, personal and social services) perspective in the primary analysis, and from the societal prospective in a sensitivity analysis.

The primary effectiveness outcome will be quality adjusted life years (QALYs) measured over a time horizon of 1 year based on EQ-5D scores collected during the trial; secondary analysis will examine the IWQoL to inform non preference-based cost-effectiveness analyses. The following cost components will be included in the analysis: weight-related inpatient and outpatient hospital, primary care and child and adolescent mental health services contacts; any other NHS treatments, or treatment paid for by the PCT, received to support weight loss; the cost of additional support provided at school; benefit receipt, family and social fund support, and the receipt of direct payments from social services or registered charities; and the impact of child care on parents' ability to work. Cost-effectiveness will be measured based on the incremental cost per QALY gained of the intervention versus the control. We shall use non-parametric bootstrap estimation to derive 95% confidence intervals for mean cost and effect differences between the trial groups and to calculate 95% confidence intervals around the incremental cost effectiveness ratios.

A series of univariate, multi-way and probabilistic sensitivity analyses will be undertaken to explore the implications of uncertainty on the incremental cost-effectiveness ratios and to consider the broader issue of the generalisability of the study results. The sensitivity analyses will include extending the baseline cost-effectiveness model to a life-time time horizon using Markov modelling. We will construct cost-effectiveness acceptability curves constructed using the net benefits approach.

## Discussion

Since receiving funding and ethical approval the intervention team have developed a comprehensive 5 day training programme for the providers. This has included training in good clinical practice and the background to obesity causes and consequences. Risk management and child protection training has also been included. Following training in specific motivational interviewing techniques and the use of solution focused questioning the providers have been trained to deliver the 12 modules through a combination of description, small group work and role play. A manual is used to enable delivery in a standardised manner by all the providers. After completion of the training all providers have observed a trained clinician delivering the intervention and have been observed delivering their first sessions by the team clinical psychologist. Self assessment checklists, live observations and audio recordings are used to record adherence to the programme using a Fidelity Adherence Scale to assess fidelity to delivery of session components and adherence to the psychological model. Providers also receive regular clinical supervision.

The prewritten standardised educational session has been delivered to Control subjects by practice nurses. We have piloted and refined a questionnaire to monitor fidelity of information giving and whether practice nurses have experience or training in motivational or other techniques.

The Chief Investigators (DC and RV) ensure that there are monthly trial management meetings with the research and intervention teams and we have been developing working relationships with GP practices and community resources in order to deliver the intervention in convenient locations for families.

As the project has been developing we have developed transparent operating procedures to ensure consistency of response to drop outs or families failing to attend intervention sessions.

Both Trial Steering and Data Monitoring and Ethics Committees have been appointed and the first meetings with the team have been completed.

### Trial Status

The HELP study is a 51 month study.

The Set-up phase took place in Months 1-6. The providers were recruited and trained to deliver the intervention and control arms in M 6-9. Recruitment of young people commenced in M9 and will continue till M33. Baseline assessments commenced in M12 and will continue for 2 years till M36. Subjects were randomised in M12, and delivery of intervention and control arms will run from M12 to M 42. Final data collection will occur M18 to 42. Database completion, analysis and writing up will occupy M45-51.

## Competing interests

AK is also Director of Public Health Strategy and Medical Director at the Health Protection Agency (HPA). The views expressed here, however, are personal and are not intended to represent the views of the HPA.

## Authors' contributions

The trial was conceptualised by DC and RV. The intervention was developed by DC with input from RV. RV and DC developed the trial design, with input from SKinra, IW, IN and TC. TC and US developed the statistical plan, with input from RV. AK, AM and LH contributed to trial design and writing of the protocol. SM and SKarlsen contributed to the trial design developed the economic evaluation plan. All authors read and approved the final manuscript.
